# Autoantibodies in COVID‐19 convalescent plasma donors

**DOI:** 10.1111/vox.70140

**Published:** 2025-10-29

**Authors:** Katerina Jazbec Gradišar, Klemen Žiberna, Polonca Mali, Ivica Marić, Primož Rožman, Elvira Maličev

**Affiliations:** ^1^ Blood Transfusion Centre of Slovenia Ljubljana Slovenia; ^2^ Biotechnical Faculty University of Ljubljana Ljubljana Slovenia

**Keywords:** anti‐SARS‐CoV‐2 antibodies, autoantibodies, COVID‐19 convalescent plasma, transfusion

## Abstract

**Background and Objectives:**

Although COVID‐19 convalescent plasma (CCP) has been successfully used to treat several viral infections, its effectiveness in neutralizing severe acute respiratory syndrome coronavirus 2 (SARS‐CoV‐2) and its broader immunological safety remain debated. Since viral infections can trigger autoimmunity, particularly through molecular mimicry and immune dysregulation, there is growing interest in understanding whether CCP contains autoantibodies that could affect recipient safety.

**Materials and Methods:**

In this study, we evaluated the presence of 20 different autoantibodies, along with anti‐SARS‐CoV‐2 antibodies, in plasma samples from CCP donors. Samples were collected at three time intervals following COVID‐19 symptom onset: 0–60, 60–120 and 120–180 days. Results were compared with those from healthy control plasma donors.

**Results:**

Several autoantibodies— anti‐Smith (anti‐SM), anti‐Sjögren syndrome antigen A, 60‐kDa isoform (anti‐SSA/Ro60), anti‐proliferating cell nuclear antigen (anti‐PCNA), anti‐Ribosomal P and anti‐ribonucleoprotein/Smith antigen complex (anti‐RNP/SM)—showed modestly elevated levels in CCP samples collected within 0–60 days post symptom onset. In the 60–120‐day period, autoantibody levels declined and approached levels observed in the control group. In the 120–180‐day period, most autoantibody levels remained comparable to control levels. Our analysis showed no significant correlations between levels of neutralizing SARS‐CoV‐2 antibodies and autoantibody concentrations nor significant differences in autoantibody profiles between donors with high and low neutralizing antibody (NAb) titres.

**Conclusion:**

Our findings suggest that SARS‐CoV‐2 infection may transiently elevate certain autoantibodies in CCP donors. However, most autoantibody levels decline over time and the overall profiles do not indicate sustained autoreactivity. These results support the immunological safety of CCP, particularly when collected between 60 and 120 days post infection, and highlight the importance of timing in optimizing both efficacy and safety in plasma donation strategies.

**Highlights**

- Transient autoantibody elevations were detected in COVID‐19 convalescent plasma (CCP) donors within 0–60 days post symptom onset, with modest increase in the levels of anti‐Smith (anti‐SM), anti‐Sjögren syndrome antigen A, 60‐kDa isoform (anti‐SSA/Ro60), anti‐proliferating cell nuclear antigen (anti‐PCNA), anti‐Ribosomal P and anti‐ribonucleoprotein/Smith antigen complex (anti‐RNP/SM) compared to controls.
- Autoantibody levels declined over time, approaching control values by 60–120 days post infection and remaining comparable at 120–180 days, indicating no sustained autoreactive profile.
- No association was found between neutralizing antibody (NAb) titres and autoantibody levels, supporting the immunological safety of CCP and underscoring the importance of donation timing for optimal efficacy and safety.

## INTRODUCTION

Severe acute respiratory syndrome coronavirus 2 (SARS‐CoV‐2) is a strain of coronavirus that causes the respiratory infection COVID‐19. The immune response following infection leads to the production of neutralizing anti‐SARS‐CoV‐2 antibodies, which inhibit viral replication and further infection of host cells in over 95% of patients (99.5% at 6 months) [1, 2]. Although neutralizing antibodies (NAbs) protect against infection, growing evidence suggests that dysregulated humoral immunity contributes to the characteristic immunopathology of COVID‐19 [3].

Infectious diseases have long been associated with the induction of autoimmune conditions, primarily through mechanisms such as molecular mimicry [4], as well as the activation of antigen‐presenting cells that can stimulate autoreactive T cells, resulting in the production of pro‐inflammatory mediators and subsequent tissue damage [5]. SARS‐CoV‐2 infection has been shown to trigger the development of autoimmunity—according to some authors—as a result of cross‐reactivity between viral antigens and host autoantigens [6]. The pathogenesis of autoimmune diseases involves the formation of various autoantibodies, which have already been detected in patients with COVID‐19 [3].

COVID‐19 thus shares several clinical features with autoimmune diseases, including arthralgia, myalgia, fatigue, keratoconjunctivitis sicca and dermatological manifestations [7, 8]. In some cases, autoimmune conditions such as systemic lupus erythematosus (SLE), haemolytic anaemia and Kawasaki disease in children have been observed in association with SARS‐CoV‐2 infection [9]. These clinical findings suggest that SARS‐CoV‐2 may induce autoimmune and/or autoinflammatory dysregulation, which may manifest during the acute phase of illness or persist as part of the post‐acute sequelae of SARS‐CoV‐2 infection, commonly referred to as long COVID [5, 8, 10].

One of the therapeutic approaches used in the COVID‐19 pandemic was the administration of COVID‐19 convalescent plasma (CCP) derived from recovered patients, which provides passive immunity and can help prevent clinical deterioration during the acute phase of infection [11, 12, 13, 14]. Apheresis of plasma from recovered donors yields a broad spectrum of antibodies and other immunomodulatory proteins that can attenuate the inflammatory response induced by SARS‐CoV‐2 [15]. A critical factor in CCP therapy is the timing of administration, which should occur as early as possible, within the first 3–9 days of symptom onset, as early high‐titre plasma has been shown to reduce viral load, progression to severe disease, hospitalization and mortality [16, 17, 18, 19]. However, current guidelines recommend administering CCP only to immunocompromised patients [20].

In addition to anti‐SARS‐CoV‐2 antibodies, CCP may also contain autoantibodies, which can affect the recipient's immune response and may partially explain the variable efficacy of CCP therapy in COVID‐19 patients [3]. Understanding the mechanisms of virus‐induced autoantibody formation and potentially implementing routine screening of donated plasma for harmful autoantibodies is increasingly important in light of the ongoing and potential emergence of new viral diseases.

Given the potential involvement of autoimmunity in COVID‐19 and the therapeutic use of convalescent plasma, this retrospective study aimed to characterize the autoantibody profile in CCP donors. Specifically, we measured the levels of 20 clinically relevant autoantibodies in plasma samples from COVID‐19 convalescent donors and compared them with those in non‐convalescent, anti‐SARS‐CoV‐2 seronegative blood donors. Additionally, we examined the relationship between autoantibody levels, anti‐SARS‐CoV‐2 antibody titres and the time elapsed from symptom onset to plasma donation.

## MATERIALS AND METHODS

### Convalescent and healthy control plasma donors

As part of the EU Emergency Support Instrument (ESI) for collecting CCP and building capacity in plasma collection within EU Member States, European Commission, Directorate‐General for Health and Food Safety, Health Systems, Medical Products and Innovation (1 September 2020–31 August 2021), 149 plasma samples were included in our study (101 CCP donors and 48 non‐convalescent, anti‐SARS‐CoV‐2 seronegative blood donors). All donors had to meet the minimum expert guidelines for plasma donations (in summary: aged 18–65 years, body weight >50 kg, general good health). All donations were collected between June 2020 and August 2021 with the apheresis procedure (SMART CONNECT PLASMACELL‐C6R2278, Fresenius Kabi, Germany). All samples for biomarker analyses were obtained from the tubes connected to the infusion bags and immediately stored at −80°C until analysis, having undergone only a single freeze–thaw event.

### SARS‐CoV‐2 antibody testing

Abbott SARS‐CoV‐2 IgG II Quant (Abbott Ireland), a second‐generation chemiluminescent microparticle immunoassay (CMIA) for the quantitative determination of IgG Abs to the receptor‐binding domain (RBD) of the S1 subunit of the SARS‐CoV‐2 spike protein, including the NAbs, was performed in the 101 CCP samples. All tests were performed according to the manufacturer's instructions.

A standard live SARS‐CoV‐2 microneutralization assay was used for NAb testing. The assay readout was the cytopathic effect, where the assay cut‐off titre was <1:20. Assay results below the lower limit of quantitation (LLQ) were set to 0.5 times the LLQ. The neutralization tests were performed on 67 CCP samples by the Institute of Microbiology and Immunology, Faculty of Medicine, University of Ljubljana. Since the primary operational goal of our centre was to collect plasma with high levels of NAbs, the samples that showed a higher likelihood of elevated titres—based on preliminary screening with the Abbott SARS‐CoV‐2 IgG II Quant assay—were submitted for neutralization testing [14].

### Detection of autoantibody levels in plasma specimens

Plasma samples were analysed using the MILLIPLEX Human Autoimmune Autoantibody Panel (#HAIAB‐10K; Merck EMD Millipore, Billerica, MA, USA) on a Luminex 200 detection instrument operated with xPONENT Software V4.2 (Luminex Corp., Austin, TX, USA). The presence of antibodies to the following 20 different antigens was determined: C1q (complement component 1q), CENP‐A (centromere protein A), CENP‐B (centromere protein B), β2‐glycoprotein, Jo‐1 (histidyl‐tRNA synthetase), Ku (Ku antigen), Mi‐2 (nucleosome remodelling deacetylase complex antigen), MPO (myeloperoxidase), PCNA (proliferating cell nuclear antigen), PL‐12 (alanyl‐tRNA synthetase), PM/Scl‐100 (100‐kDa subunit of the PM/Scl complex), PR3 (proteinase 3), Ribosomal P, RNP (small nuclear ribonucleoprotein), RNP/Sm (ribonucleoprotein/Smith antigen complex), Scl‐70 (DNA topoisomerase I), Sm (Smith antigen), SSB/La (Sjögren syndrome antigen B, or La protein), SSA/Ro52 (Sjögren syndrome antigen A, 52‐kDa isoform) and SSA/Ro60 (Sjögren syndrome antigen A, 60‐kDa isoform). The selection of the autoantibodies was based on their clinical relevance and frequent inclusion in standard autoimmune diagnostic panels. These autoantibodies are commonly associated with systemic autoimmune diseases (such as SLE, Sjögren's syndrome and systemic sclerosis) and represent key targets involved in autoimmune processes potentially triggered by viral infections.

### Statistical analysis

The dataset contained measurements for multiple autoantibodies from plasma samples of 149 individuals. We excluded the measurements where the relative error of the technical replicates exceeded 30%. Data are presented as the mean and standard error of the mean for normally distributed data, median and interquartile range (IQR) for non‐parametric data, the count and the percentage for binary data and as a geometric mean with 95% confidence interval for neutralization test titre values. Statistical comparison between the two groups was performed using the Student *t*‐test for normally distributed data, the Mann–Whitney *U* test for non‐parametric data and the Fisher exact test for binary data. Multiple group comparison was performed using the Kruskal–Wallis ANOVA test.

The heatmaps of correlated autoantibodies were generated by standard scaling of autoantibody measurements, followed by calculating correlations between each other. Autoantibodies were then hierarchically clustered using the Ward variance minimization algorithm.

The analysis was performed using Python 3.12, NumPy 2.3, Pandas 2.3, SciPy 1.5, SciKit Learn 1.7 and Statsmodels 0.14.

### Ethics statement

This study was performed under the approval of the National Medical Ethics Committee of the Republic of Slovenia (0120‐241/2020‐8, from 18 June 2020). All volunteers provided written informed consent before enrolment and sample provision.

## RESULTS

### Study participant characteristics

The analysis was conducted on the data of 101 CCP donors and 48 non‐convalescent control donors. More than 89% of the plasma donors from both groups were males. The median age of all plasma donors was 42 (20–62 years old); CCP donors were slightly older (45 years) than the control donors (40 years). CCP donors exhibited blood groups O (36%), A (35%), B (19%) and AB (11%), and the control donors had blood groups O (42%), A (41%), B (8%) and AB (8%). The median period from the end of infection symptoms to plasma collection was 108 days, with the shortest time from recovery being 31 days. A majority of the donors had had mild to moderate COVID‐19 disease. A SARS‐CoV‐2 IgG antibody test was performed for all donors. The neutralization antibody test was performed for 67 CCP donors (66%).

### Autoantibody measurements in CCP donors and non‐convalescent plasma donors

**FIGURE 1:**
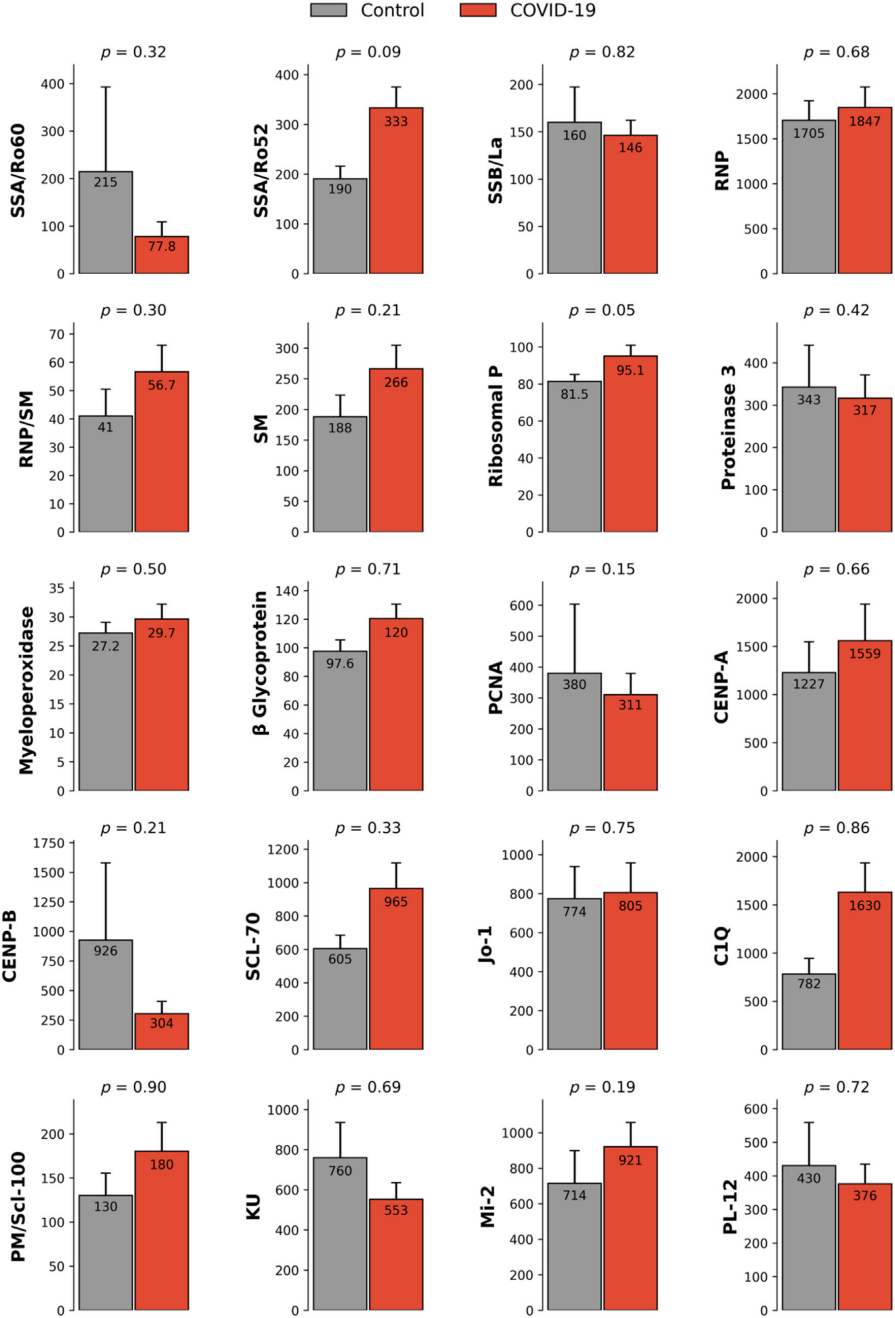
Autoantibody comparison against common antigens between COVID‐19 convalescent plasma (CCP) donors and non‐convalescent donors. Comparison of autoantibody measurements between CCP donors (*N* = 101) and controls (*N* = 48). CCP was collected from 0 to 180 days after the start of COVID‐19 symptoms. All samples were analysed in duplicates by Luminex technology. C1q, complement component 1q; CENP‐A, centromere protein A; CENP‐B, centromere protein B; Jo‐1, histidyl‐tRNA synthetase; Ku, Ku antigen; Mi‐2, nucleosome remodeling deacetylase complex antigen; MPO, myeloperoxidase; PCNA, proliferating cell nuclear antigen; PL‐12, alanyl‐tRNA synthetase; PM/Scl‐100, 100 kDa subunit of the PM/Scl complex; PR3, proteinase 3; RNP, small nuclear ribonucleoprotein; RNP, Ribosomal P; RNP/Sm, ribonucleoprotein/Smith antigen complex; Scl‐70, DNA topoisomerase I; Sm, Smith antigen; SSA/Ro52, Sjögren syndrome antigen A, 52 kDa isoform; SSA/Ro60, Sjögren syndrome antigen A, 60 kDa isoform; SSB/La, Sjögren syndrome antigen B, or La protein.

Multiplex Luminex analysis evaluated a panel containing antibodies against 20 antigens (C1q, CENP‐A, CENP‐B, β2‐glycoprotein, Jo‐1, Ku, Mi‐2, MPO, PCNA, PL‐12, PM/Scl‐100, PR3, Ribosomal P, RNP, RNP/Sm, Scl‐70, Sm, SSB/La, SSA/Ro52 and SSA/Ro60). The comparison between control and CCP samples did not reveal any statistically significant differences, as shown in the graphs in Figure 1. Several autoantibodies, including Ribosomal P (*p =* 0.05), SSA/Ro52 (*p =* 0.094) and PCNA (*p =* 0.154), showed modestly elevated median values in COVID‐19 samples compared to controls; however, only Ribosomal P reached statistical significance. Conversely, anti‐CENP‐B, anti‐Jo‐1 and anti‐Scl‐70 were slightly lower in the COVID‐19 group, although these differences were also not statistically significant. The broad IQRs and overlapping distributions indicate high inter‐individual variability and no consistent pattern of autoantibody elevation following SARS‐CoV‐2 infection (Figure 1).

### Changes in plasma autoantibody measurements in CCP donors and non‐convalescent control plasma donors over time

**FIGURE 2:**
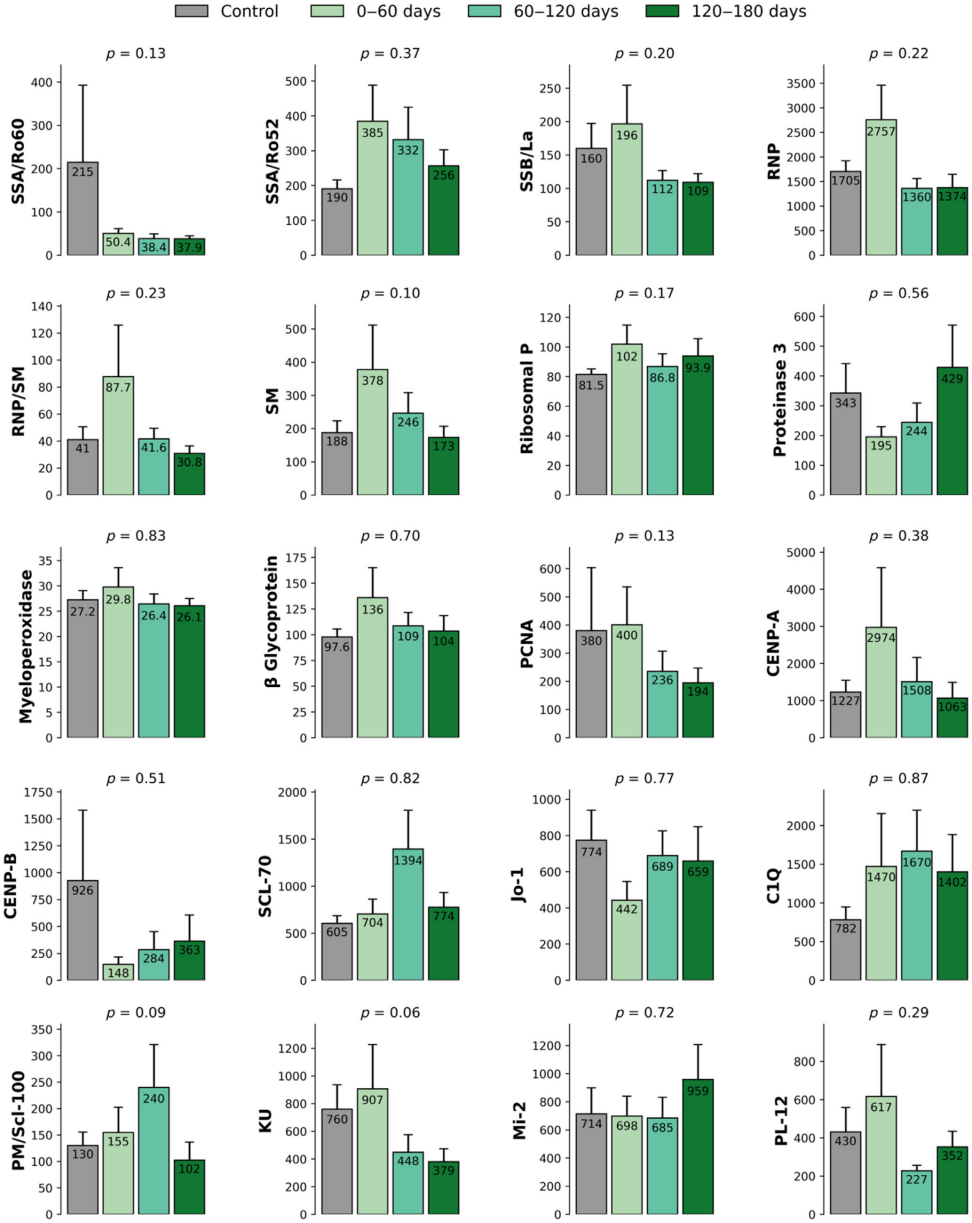
Autoantibody levels against common autoantigens in COVID‐19 convalescent plasma (CCP) donors and non‐convalescent donors, stratified by time since symptom onset. Autoantibody levels in CCP individuals grouped by different time intervals after the start of COVID‐19 symptoms to donation (0–60 days [15.8% CCP donors], 60–120 days [34.7% CCP donors] and 120–180 days [32.7% CCP donors]). All plasma samples were analysed in duplicates by Luminex technology. C1q, complement component 1q; CENP‐A, centromere protein A; CENP‐B, centromere protein B; Jo‐1, histidyl‐tRNA synthetase; Ku, Ku antigen; Mi‐2, nucleosome remodeling deacetylase complex antigen; MPO, myeloperoxidase; PCNA, proliferating cell nuclear antigen; PL‐12, alanyl‐tRNA synthetase; PM/Scl‐100, 100 kDa subunit of the PM/Scl complex; PR3, proteinase 3; RNP, small nuclear ribonucleoprotein; RNP, Ribosomal P; RNP/Sm, ribonucleoprotein/Smith antigen complex; Scl‐70, DNA topoisomerase I; Sm, Smith antigen; SSA/Ro52, Sjögren syndrome antigen A, 52 kDa isoform; SSA/Ro60, Sjögren syndrome antigen A, 60 kDa isoform; SSB/La, Sjögren syndrome antigen B, or La protein.

To evaluate potential time‐dependent differences in autoantibody levels, CCP donors were divided into three groups based on the time elapsed since the onset of COVID‐19 symptoms to donation: Group A (0–60 days), Group B (60–120 days) and Group C (120–180 days). As shown in Figure 2, a modest increase in autoantibody levels was observed in the early phase (0–60 days) compared to controls. Between days 60 and 120, levels generally declined and approached those seen in the control group. In the final interval (120–180 days), most autoantibody levels remained stable, with some markers showing minor increases or decreases. However, none of these temporal differences reached statistical significance, indicating a lack of consistent, time‐dependent trends in circulating autoantibody levels following COVID‐19.

During the 0–60‐day period following the onset of COVID‐19 symptoms, several autoantibodies showed modestly elevated levels in CCP compared to controls. The most notable differences were observed for anti‐SM (*p =* 0.036), anti‐SSA/Ro60 (*p =* 0.059), anti‐PCNA (*p =* 0.075), anti‐Ribosomal P (*p =* 0.078) and anti‐RNP/SM (*p =* 0.079). These findings suggest a possible transient early increase in these autoantibodies, although the results should be interpreted with caution because of the multiple comparisons made and the overall heterogeneity of the responses (Figure 2).

In the 60–120‐day period, autoantibody levels in most convalescent donors declined and approached levels observed in the control group. No autoantibodies showed significant differences compared to controls during this interval.

In the 120–180‐day period, no statistically significant differences in autoantibody levels were observed between CCP and controls. Most autoantibodies, including anti‐SSA/Ro60, anti‐SSA/Ro52, SM, anti‐Ribosomal P and anti‐PCNA, remained comparable to control levels.

### Correlations between autoantibodies

**FIGURE 3:**
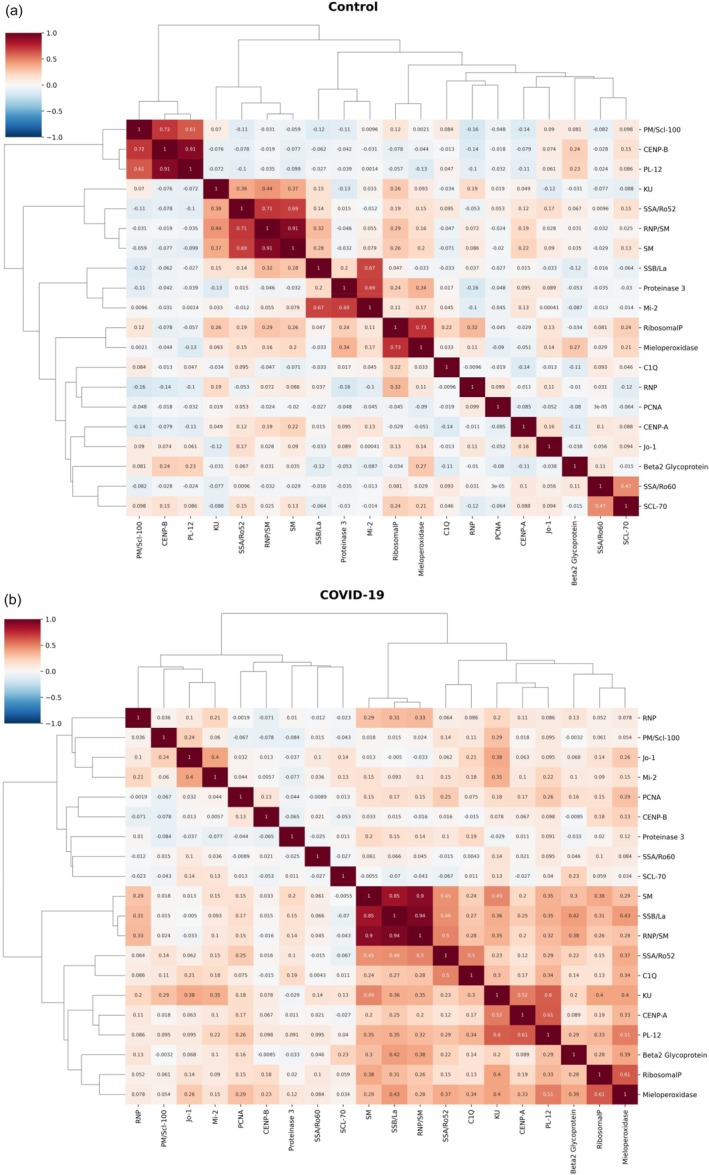
Heatmap of autoantibodies against common autoantigens and dendrogram analysis. Correlation matrix heatmaps and hierarchical clustering dendrograms representing associations among autoantibodies in the control donor group (a) and the COVID‐19 convalescent plasma (CCP) group (b). In controls, only moderate correlations were observed, mainly among anti‐ribonucleoprotein/Smith antigen complex (anti‐RNP/SM), anti‐Smith (anti‐SM), anti‐centromere protein B (anti‐CENP‐B), anti‐alanyl‐tRNA synthetase (anti‐PL‐12), anti‐PM/Scl‐100, anti‐CENP‐B (0.72), and anti‐Sjögren syndrome antigen A, 52‐kDa isoform (anti‐SSA/Ro52) (*r* = 0.69–0.91), indicating limited background clustering. In contrast, CCP displayed stronger and more extensive correlations, particularly involving SM, Sjögren syndrome antigen B, or La protein (SSB/La), RNP/SM and Sjögren syndrome antigen A, 52‐kDa isoform (SSA/Ro52) (*r* = 0.45–0.96). The colour scale represents correlation strength (from −1 to +1). C1q, complement component 1q; CENP‐A, centromere protein A; Jo‐1, histidyl‐tRNA synthetase; Ku, Ku antigen; Mi‐2, nucleosome remodeling deacetylase complex antigen; MPO, myeloperoxidase; PCNA, proliferating cell nuclear antigen; PM/Scl‐100, 100 kDa subunit of the PM/Scl complex; PR3, proteinase 3; RNP, small nuclear ribonucleoprotein; RNP, Ribosomal P; Scl‐70, DNA topoisomerase I; Sm, Smith antigen; SSA/Ro52, Sjögren syndrome antigen A, 52 kDa isoform; SSA/Ro60, Sjögren syndrome antigen A, 60 kDa isoform; SSB/La, Sjögren syndrome antigen B, or La protein.

Correlation analysis of autoantibody profiles revealed that there were distinct patterns between healthy control samples and CCP samples (Figure 3). In the control group, a few moderate correlations were observed, including anti‐RNP/SM and anti‐SM (*r* = 0.91), anti‐CENP‐B and anti‐PL‐12 (*r* = 0.91), anti‐PM/Scl‐100 and anti‐CENP‐B (*r* = 0.72) and anti‐RNP/SM and anti‐SSA/Ro52 (*r* = 0.71). In the COVID‐19 group, the correlations found between SM, SSB/La, RNP/SM and SS/Ro52 were stronger, forming a tightly interconnected cluster (*r* = 0.45–0.96). These findings suggest a more coordinated pattern of autoreactivity following SARS‐CoV‐2 infection.

### Comparison of plasma autoantibody concentrations with SARS‐CoV‐2 IgG and NAb titres in CCP donors

In the COVID‐19 group, we compared and evaluated the correlation between autoantibody levels and SARS‐CoV‐2 IgG antibody levels or SARS‐CoV‐2 NAb titres. CCP donors had IgG Ab levels between 13 and 28,000 (AU/mL) and NAb titres between 10 and 1280. There was no statistically significant correlation between any autoantibody and IgG Ab levels (Figure S1) or between any autoantibody and NAb titres (Figure S2). Similarly, there were no statistically significant differences between autoantibody levels of the low NAb titre (<160) and high NAb titre (>160) groups. The only exception approaching significance was β2 glycoprotein, which showed higher levels in the high NAb titre group (67.5 [43.0–142.25] [*N* = 43] vs. 110.0 [91.75–146.75] [*N* = 23], *p =* 0.054) (Figure S3). This suggests a possible link between stronger antiviral humoral responses and transient autoantibody production, highlighting the need to better understand how immune activation might contribute to autoreactivity.

## DISCUSSION

CCP collected from individuals who have recovered from SARS‐CoV‐2 infection was used during the COVID‐19 pandemic as a passive immunotherapy for hospitalized patients with severe disease [15]. CCP contains NAbs capable of reducing viral infectivity and dampening the inflammatory response [21]. However, there has been increasing concern that transfusion of plasma with an unfavourable autoantibody profile may be associated with adverse clinical outcomes [22]. Our study directly addresses this concern by analysing the presence and dynamics of 20 autoantibodies in CCP from recovered donors and by comparing them with non‐convalescent blood donor controls.

Our findings show that although average concentrations of several autoantibodies were higher in CCP samples than in controls, most differences did not reach statistical significance, except for anti‐Ribosomal P autoantibodies (*p =* 0.05). In subgroup analyses of CCP samples collected within 0–60 days of symptom onset, we observed significantly higher levels of anti‐SM antibodies (*p =* 0.036) and trends towards elevated levels of anti‐SSA/Ro60 (*p =* 0.059), anti‐SSA/Ro52 (*p =* 0.086), anti‐Ribosomal P (*p =* 0.078), anti‐PCNA (*p =* 0.075), anti‐SSB/La (*p =* 0.128), anti‐PM/Scl‐100 (*p =* 0.301) and anti‐Ku (*p =* 0.147) antibodies. These autoantibodies have known associations with systemic autoimmune conditions: anti‐SSA/Ro60 and anti‐SSB/La are classically associated with Sjögren's syndrome and SLE; elevated anti‐SM antibodies are highly specific for SLE; anti‐SSA/Ro52 antibodies are associated with several autoimmune conditions such as rheumatic diseases, inflammatory myositis and autoimmune liver disease; and anti‐Ku antibodies are found in mixed connective tissue disease and systemic sclerosis [23, 24, 25, 26, 27].

Anti‐Ribosomal P antibodies, which are strongly associated with neuropsychiatric manifestations of SLE, including depression and psychosis [28, 29], were found to be significantly elevated when all CCP samples were analysed collectively, although this significance did not remain statistically significant when samples were stratified by time intervals (0–60, 60–120 and 120–180 days post symptom onset; *p =* 0.12). This elevation is particularly noteworthy given that these donors had no known autoimmune disease and also reported no symptoms at the time of plasma donation, suggesting a possible subclinical autoimmune activation. However, the lack of statistical significance across individual time intervals may indicate variability among donors or that anti‐Ribosomal P elevation is transient or confined to a subset of individuals.

The temporary elevation of all these autoantibodies in CCP suggests that SARS‐CoV‐2 infection may transiently break immune tolerance, mimicking autoimmunity in a subset of individuals, even in the absence of clinical symptoms. These observations are consistent with reports of transient autoantibody induction following SARS‐CoV‐2 infection as documented in studies such as those by Acosta‐Ampudia et al., which described persistent autoantibody presence and immune activation in some individuals recovering from COVID‐19 [30, 31]. Most clinical manifestations reported in association with SARS‐CoV‐2–related autoimmunity have primarily been observed in patients with moderate to severe COVID‐19 or in patients with long COVID‐19 syndrome. While autoantibody production seems to develop early during the acute phase of COVID‐19, autoimmune manifestations can occur weeks or months later when the symptoms of infection have disappeared and the patient has recovered [32].

Despite the presence of elevated autoantibodies early post infection, cross‐sectional analysis in our study revealed a general trend of decline over time. Between 60 and 120 days post symptom onset, autoantibody levels typically decreased to near‐control levels. In the final 120–180‐day interval, no clear trend was observed—some autoantibodies declined further while others showed slight rebounds. This pattern mirrors reports from longitudinal studies that found delayed or fluctuating declines in autoantibody titres post COVID‐19 [33]. Even though this might indicate ongoing immune dysregulation in a subset of individuals, we observed no statistically significant differences in this later phase. Son et al. who studied COVID‐19 patients with varying acute‐phase severities, reported persistence of certain autoantibodies for up to 1 year after infection, among them anti‐U1‐snRNP, anti‐SSB/La and anti‐PM‐Scl [33].

Our autoantibody analysis of healthy CCP donors who had mild to moderate symptoms during infection showed no significant correlations between levels of neutralizing SARS‐CoV‐2 antibodies and autoantibody concentrations. Additionally, there were no significant differences in autoantibody profiles between donors with high and low NAb titres. This suggests that the presence of neutralizing immunity does not necessarily increase the risk of autoimmunity in COVID‐19 convalescent donors. Notably, the observed borderline elevation of β2‐glycoprotein in the high‐NAb titre group warrants further investigation, particularly in the context of post‐COVID thrombotic phenomena and potential cross‐reactive responses. However, the lack of broad autoantibody induction across the panel reinforces the immunological safety of high‐titre CCP donations.

From a transfusion safety perspective, our findings are reassuring. Across all 20 autoantibodies analysed, there were no sustained or widespread elevations that would suggest a consistent risk of transmitting autoreactivity through CCP. This aligns with large safety studies showing low adverse event rates in CCP‐treated patients [34]. However, our results also emphasize that the timing of plasma collection is critical. Plasma collected too early (e.g., <60 days post symptoms) may still carry transiently elevated autoantibodies, while collection too late (e.g., >120 days) may result in insufficient NAb titres for therapeutic efficacy. Therefore, optimizing the CCP donation window—likely between 60 and 120 days post symptom onset—may offer the best balance between minimizing autoantibody content and maximizing antiviral potency. This insight is particularly important for establishing safe and effective plasma donation protocols in future outbreaks involving immune‐activating pathogens.

We acknowledge several limitations of our study. A larger sample size would have increased statistical power, especially in subgroup comparisons. A longitudinal sampling design would have allowed us to track individual‐level changes in autoantibody levels over time. Additionally, access to pre‐infection samples would have enabled us to distinguish between pre‐existing and infection‐induced autoantibodies.

In conclusion, our study provides valuable evidence that while SARS‐CoV‐2 infection can transiently elevate certain autoantibodies, CCP collected during the appropriate time window remains a safe and immunologically stable option for passive immunotherapy. These findings support continued CCP collection from mild to moderate COVID‐19 survivors and highlight the need for time‐sensitive screening strategies to ensure therapeutic quality and safety in epidemic response efforts.

## CONFLICT OF INTEREST STATEMENT

The authors declare no conflicts of interest.

## Supporting information


**Figure S1.** Correlation between autoantibodies and Abbott quantitative SARS‐CoV‐2 antibodies.
**Figure S2**. Correlation between autoantibodies and neutralization test titre values.
**Figure S3**. Autoantibody levels against common autoantigens in the low neutralizing antibody (NAb) titre (<160) and high NAb titre (>160) groups.

## Data Availability

The data that support the findings of this study are available from the corresponding author upon reasonable request.
