# Supplementary material for: Autoantibodies in COVID‐19 convalescent plasma donors
Source: Vox Sang. 2025 Oct 29;121(1):54–63. doi: 10.1111/vox.70140 (PMC12803771; doi:10.1111/vox.70140)
Supplement: Supplementary file 1 — Figure S1. Correlation between autoantibodies and Abbott quantitative SARS‐CoV‐2 antibodies. Figure S2. Correlation between autoantibodies and neutralization test titre values. Figure S3. Autoantibody levels against common autoantigens in the low neutralizing antibody (NAb) titre (<160) and high NAb titre (>160) groups. [file VOX-121-54-s001.docx]

**Supplementary Figure S1:** Correlation between autoantibodies and Abbott quantitative SARS-CoV-2 antibodies.





**Supplementary Figure S2:** Correlation between autoantibodies and neutralization test titer values.


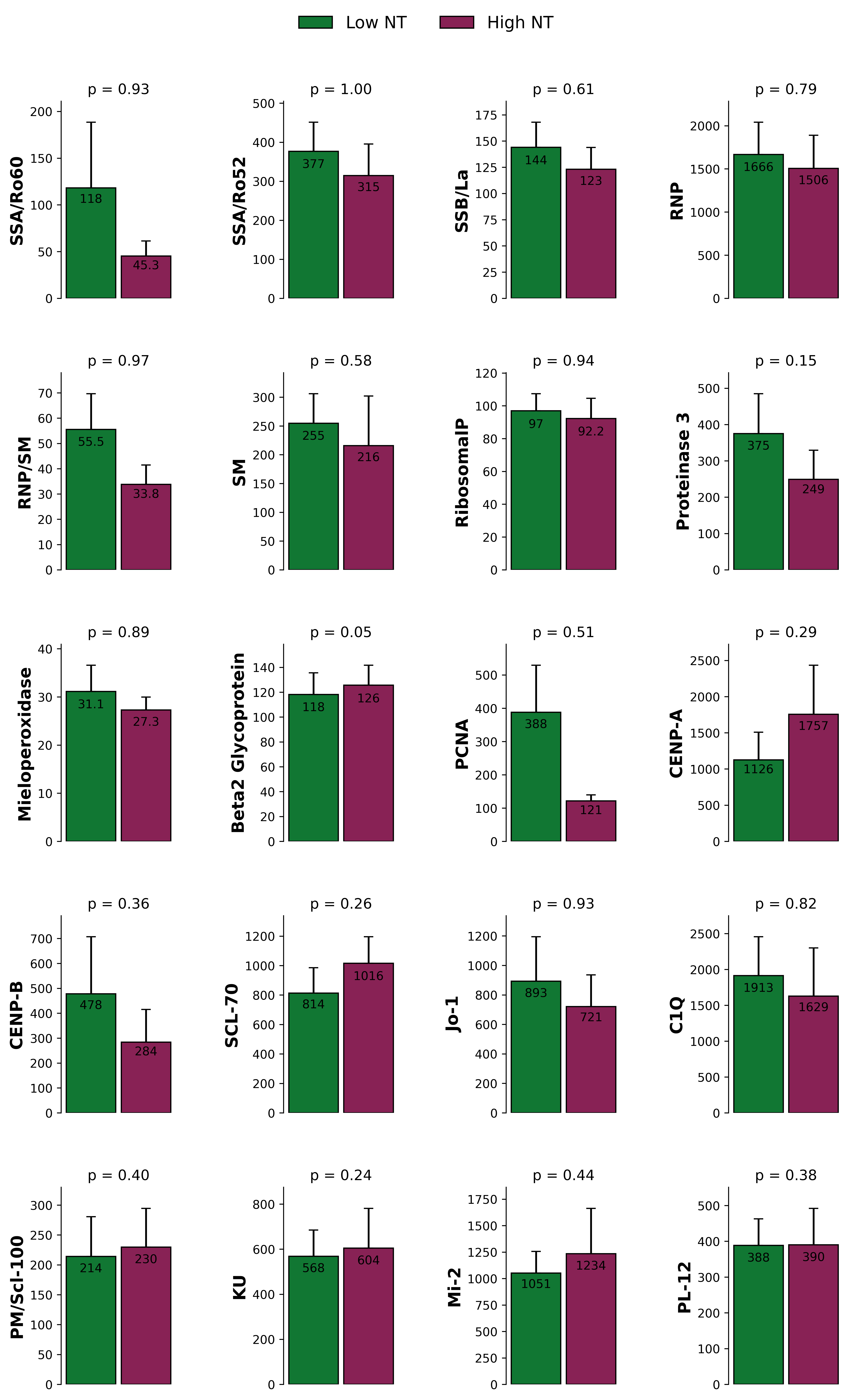


**Supplementary Figure S3**: Autoantibody levels against common autoantigens in the low NAb titer (<160) and high NAb titer (>160) groups
